# Perioperative Anti-Fibrotic Treatment Prevents Acute Exacerbation of Idiopathic Pulmonary Fibrosis After Lung Cancer Surgery

**DOI:** 10.3390/life14111506

**Published:** 2024-11-19

**Authors:** Stefano Bongiolatti, Alberto Salvicchi, Elisabetta Rosi, Elena Bargagli, Giovanni Mugnaini, Alessandro Gonfiotti, Federico Lavorini, Paolo Spagnolo, Andrea Dell’Amore, Federico Rea, Luca Voltolini

**Affiliations:** 1 Thoracic Surgery Unit, Careggi University Hospital, 50134 Florence, Italyg.mugnaini12@gmail.com (G.M.); luca.voltolini@unifi.it (L.V.); 2 Respiratory Medicine Unit, Careggi University Hospital, 50134 Florence, Italy; 3 Respiratory Diseases, Azienda Ospedaliero-Universitaria Senese, University of Siena, 53100 Siena, Italy; 4 Department of Experimental and Clinical Medicine, University of Florence, 50134 Florence, Italy; 5 Section of Respiratory Diseases, Padua University Hospital, 35128 Padua, Italy; paolo.spagnolo@unipd.it; 6 Division of Thoracic Surgery, Padua University Hospital, 35128 Padua, Italy

**Keywords:** anti-fibrotic treatment, pirfenidone, nintedanib, lung surgery, acute exacerbation

## Abstract

Background: The surgical treatment of concomitant lung cancer in patients with idiopathic pulmonary fibrosis is challenging due to the risk of life-threatening complications such as acute exacerbation development in the perioperative period. Few studies have investigated the role of anti-fibrotic drugs in this setting. The aim of this multicenter retrospective study was to evaluate the incidence of acute exacerbation, according to Collard, after lung resection in patients affected by concomitant idiopathic pulmonary fibrosis and lung cancer who were or were not on antifibrotic treatment. Secondary outcomes included: 30 and 90-day mortality and an estimation of overall and disease-free survival. Material and Methods: The study population consisted of patients affected by idiopathic pulmonary fibrosis who received curative-intent lung surgery in three Italian academic centers between 2015 and 2022. Patients were divided into two groups based on whether they were on perioperative treatment with anti-fibrotic drugs (chronical or prophylactic use) or not. To define predictors of acute exacerbation, univariate and multivariable exact logistic regression analysis were performed. The Kaplan–Meier method with log-rank test was used to estimate survival. Results: During the study period, *n* = 55 patients underwent lung resection for lung cancer, including 29 patients who were treated with antifibrotic agents. Although the sample size was small and few events were studied, the incidence of acute exacerbation was significantly lower among patient on anti-fibrotic therapy (3.4% vs. 23.1%, *p* = 0.044); in addition, anti-fibrotic treatment was the strong factor preventing acute exacerbation at the multivariable analysis (OR 0.089, *p* = 0.038). Post-operative 30- and 90-day mortality rates were not significantly lower in the anti-fibrotic treatment group (0% and 0% vs. 7.7% and 11.5%, *p* = 0.21 and *p* = 0.099, respectively). Overall and disease-free survival rates were similar. Conclusions: Considering the limitations of this retrospective study with a small sample size, anti-fibrotic perioperative treatment was associated with reduced incidence of acute exacerbation. Based on these real-world data, this pathway could be proposed as a prophylactic treatment in patients with concomitant idiopathic pulmonary fibrosis and cancer undergoing lung resection.

## 1. Introduction

Interstitial lung diseases (ILD) are a heterogeneous group of chronic, progressive and often lethal pulmonary diseases that may be complicated by lung cancer (LC) [1,2] and episodes of acute worsening referred to as acute exacerbation (AE) [3]. AE-ILD, which may occur following surgical, radiotherapeutic or chemotherapeutic treatment, is a life-threatening event with post-operative incidence and mortality ranging between 4.2 and 12.4% and 33–100%, respectively [4,5,6,7,8]. Several predictive factors of AE-ILD development have been identified including sex being male, a vital capacity (VC) < 80%, lobectomy or pneumonectomy, previous history of AE, pre-operative steroid use, high levels of Krebs von den Lungen-6 (KL-6) and usual interstitial pneumonia (UIP) patterns in computed tomography (CT) scans [7,8].

Among the ILDs, idiopathic pulmonary fibrosis (IPF) has worse outcomes than other ILD subtypes, and surgery for concomitant LC is particularly challenging, exposing the patient to a high risk of AE and a consequently high post-operative mortality. Moreover, ILD patients, particularly IPF patients, also had significantly reduced long-term survival rates when compared to patients without ILD who were operated on for lung cancer [7,9,10,11].

The current treatment of IPF, based on anti-fibrotic drugs (AFDs), pirfenidone and nintedanib, has demonstrated reductions in the decline of pulmonary function over time [12,13], disease progression and the incidence of AE [14], but the utility of these agents in the postoperative period in reducing the incidence of AE is not known, especially in Western countries. Small retrospective series and a prospective phase II study from Japan have recently shown that perioperative pirfenidone treatment is feasible [15] and it may reduce the incidence of AE [16] and its associated mortality, suggesting a role for its prophylactic use in this setting [15,16,17]. Iwata et al. [15] reported a significant reduction in AE after surgery (21.1% vs. 3.2%) in patients treated with pirfenidone, and this datum was partially confirmed in a larger cohort study in which 90-day AE was observed in 16.7% vs. 3.6% [16]. In the prospective phase II trial (PEOPLE study) [17] AE occurred in 1 patient among the 39 patients eligible and the authors could only conclude that perioperative pirfenidone treatment was safe with a promising effect on AE. However, the current guidelines on management of AE do not recommend a preventive drug administration to protect against post-surgical AE [4], and the experience in this regard is very limited in Western countries.

The aim of this study was to evaluate the usefulness of perioperative anti-fibrotic treatment in reducing the incidence of post-operative AE (within 90 days of surgery) and its consequent associated mortality in IPF patients undergoing surgery for lung cancer.

## 2. Materials and Methods

### 2.1. Patient Population

The study population consisted of patients affected by IPF who received curative-intent lung surgery for cancer in three Italian academic hospitals (Florence, Padua, Siena). All the clinical data were collected and analyzed in a common database including only patients consecutively treated between January 2015 and December 2022. IPF was defined according to the latest international guidelines [1,18]. All patients were evaluated in each center by the Institutional Tumor Boards and the diagnosis of IPF was performed by multidisciplinary discussion at ILD Referral Center. Only patients with IPF confirmed at pathology were included in our analysis.

Patients affected by ILD different from IPF, patients undergoing lung resection for diagnostic purposes and those on long-term oxygen therapy were excluded from the study. Prophylactic perioperative AFT was applied to all patients with newly diagnosed IPF not receiving chronic AFT. We divided the study population in two groups based on the use or lack of use of perioperative AFT (pirfenidone or nintedanib). The two groups were compared in terms of demographics, pulmonary function test values, comorbid diseases, type of surgical procedure and approach, clinical and pathological LC stage, post-operative complications, length of hospital stay, length of intensive care unit (ICU) stay, overall survival and LC recurrence.

For patients with a new diagnosis of IPF associated with lung cancer, the AFT was started two to four weeks before surgery with pirfenidone at a dose of 801 mg that was increased to 2403 mg per day for at least 7–10 other days (as “prophylaxis”). For patients with a previous diagnosis of IPF with chronic treatment, the AFT was continued along the whole perioperative period, except for patients in treatment with nintedanib, which was stopped for 2–5 days before surgery and promptly re-started when the bleeding risk was nulled. After surgery the “prophylactic” AFT was continued at least for 6 months post-operatively and discontinued based on pulmonary function test values and on the evaluation of the ILD Referral Center. Chronic AFT, if well tolerated, was never discontinued. The no-treatment group consisted of patients for whom IPF was diagnosed after operation or who were referred to lung cancer surgery in the years preceding the use of prophylactic AF drugs. During the study period, perioperative and intraoperative treatments for patients with IPF and lung cancer were unchanged, except for the AFT.

In case of suspicion of post-operative AE, within 90 days after surgery, a CT scan was immediately performed and the diagnosis was confirmed in a multidisciplinary meeting with radiologists and pulmonologists. According to the definition by Collard et al. [19], AE was considered an acute, clinically significant respiratory deterioration characterized by evidence of new, widespread alveolar abnormality with the exclusion of alternative etiologies developed less than one month after curative-intent lung surgery for cancer in IPF patients.

The pre-operative functional assessment included arterial blood gas analysis and spirometry, pletysmography and measuring of the diffusing capacity of the lung for carbon monoxide and, in patients with limited lung function, a ventilation/perfusion scan and/or cardio-pulmonary exercise testing was performed. Postoperative complications and mortality, defined as any death within the 90 days following surgery, or during the same hospitalization, as well as overall survival (OS) and disease-free survival (DFS) were calculated. Local recurrence was defined as disease recurrence in the preserved lobe; regional recurrence in a homolateral lobe/s other than the preserved lobe, hilar or mediastinal lymph nodes, and distant recurrence as any metastasis developed in extra-thoracic organs, contralateral lung or ipsilateral pleura [20]. All the patients completed the follow-up and were included in the survival analysis. The last follow-up visit was in December 2022.

### 2.2. Ethical Statement

This retrospective study was reviewed and approved by the local ethics committee (CEAVC, approval number 22254_PF, 26 April 2023). In accordance with Italian laws for observational studies, waiver of informed consent was gained from study participants.

### 2.3. Statistical Analysis

Statistical analysis was performed using SPSS 24.0 (IBM SPSS Statistics for Macintosh, Version 24.0. Armonk, NY, USA). Continuous variables, expressed as median and interquartile range (IQR) were compared with Wilkoxon–Mann–Whitney test; categorical variables were analyzed using the χ^2^ test or Fisher’s exact test, as appropriate. All *p*-values were 2-sided, with statistical significance set <0.05. To define predictors for AE, a univariate and multivariable exact logistic regression analysis was performed for clinical variables that may influence this complication, selected for clinical relevance or based on previous published studies [21].

The Kaplan–Meier method was used to estimate OS and DFS. Overall survival was calculated from the date of operation to death or date of the last follow-up (December 2022); DFS was calculated from the date of surgery to the date of the first evidence of cyto-histologically proven recurrence or death. Follow-up was assessed by outpatient visits, including interval medical history, physical examination and enhanced contrast whole body-CT scan every six months. Differences in OS and DFS between groups were evaluated by log-rank test.

## 3. Results

During the study period, *n* = 55 IPF patients underwent lung resection for LC in the three academic institutions: demographic, functional and oncological pre-operative characteristics are depicted in Table 1. The AFT group included 29 IPF patients: in 15 (51.7%) patients the AFT treatment was started just before surgery as a prophylactic measure, whereas 14 (48.3%) were chronically treated with AFD and, in particular, 5 (17.3%) were treated with nintedanib and 9 (31%) with pirfenidone. The remaining 25 IPF patients constituted the control group.

The majority of patients in both groups were men (72.4% vs. 76.9%) with a median age of 72 (IQR 11) and 75.5 (IQR 12) years in the AFT group and no-treatment group, respectively. The two groups were balanced regarding most functional, demographic, comorbid diseases or operative variables except for significantly higher ECOG PS values and shorter operative times for patients in the AFT group. Moreover, in this group, the patients had a trend toward significance of lower DLCO% and SpO2 levels at rest. Squamous cell carcinoma was the most frequent cancer subtype, affecting 50% and 44.8% of the patients in the AFT group and non-treatment group. No delayed surgery or serious adverse events developed in the 15 (51.7%) AFT patients who started prophylactic AFT.

### 3.1. Post-Operative Outcomes

Post-operative results are summarized in Table 1. Detailed post-operative complication are resumed in Table 1. Major complications, classified as 3a or more according to the Clavien-Dindo classification [22], occurred in 7 (24.1%) and 8 (30.8%) patients, respectively (*p* = 0.76). However, the rate of AE was significantly different between the AFT group and the no-treatment group (3.4% vs. 23.1%, *p* = 0.044). Interestingly, the incidence of AE was 0% (0% vs. 23.1%, *p* = 0.039) in patients treated with prophylactic AFT. Thirty- and ninety-day post-operative mortality were not-significantly higher in the no-treatment group (7.7% and 11.5% vs. 0% and 0%, *p* = 0.21 and 0.09). The consequences of developing AE were as follows: high mortality (3/6 patients died), long ICU stay (median 9.5 days) and long hospital stay (26.5 days).

By adding some clinical variables, selected based on clinical relevance, into the logistic regression model (Table 2), we identified DLCO% > 55 and AFT as prognostic protective factors for developing AE on univariate analysis, but only AFT treatment was a significant prognostic factor on multivariable analysis (OR 0.089, *p* = 0.038)

### 3.2. Oncological Outcomes

At the median follow-up of 24 months (17 months for AFT group and 23.5 months for no-treatment group), *n* = 11 patients died, specifically from lung cancer relapse (*n* = 5), sequelae of neurological disorders (*n* = 2), complications of coronary artery disease (*n* = 2), respiratory failure secondary to IPF (*n* = 1) and other tumors (*n* = 1). Overall survival was similar between the two groups; 5-year OS rates were 45% vs. 58% in the AFT group and no-treatment group (*p* = 0.65). Similarly, DFS was not different between the two groups.

## 4. Discussion

The treatment of LC in patients with IPF is particularly challenging for various reasons, including impaired pulmonary function, the patients’ frailty and the increased risk of post-operative complications such as pneumonia, respiratory failure and AE, all carrying a poor prognosis. Any LC treatment can trigger AE: chemotherapy, target therapy and immunotherapy [23], radiotherapy and stereotactic ablative radiotherapy, which are associated with significant toxicities and frequently contraindicated [24], as well as surgical resection [8,9,10,11]. Surgery can trigger AE through direct lung manipulation and damage, or through the stress on the fibrotic parenchyma induced by mechanical ventilation or hyperoxia, with an incidence of AE ranging between 4.2% and 12.4% [7,8]. Acute exacerbation of ILD is a life-threatening adverse event with mortality rates of 33.3–100% [4]. Several factors have been identified as predictors [7,8], such as male gender, usual interstitial pneumonia (UIP) patterns on CT, surgical procedures or operation time, according to some laboratory data [8]. A treatment approach that could potentially reduce the incidence of AE has not been suggested or identified yet [4]. Anti-fibrotic therapies are, nowadays, the mainstay of the treatment of IPF [12], but only few retrospective studies from Asia have suggested a potential protective role for perioperative AFT [15,16,25,26].

Our multicenter study showed a significantly lower rate of 30-day AE in the cohort of patients treated with AFDs and a consequent trend towards reduced 90-day mortality in these patients (11.5% vs. 0%, *p* = 0.099). Multivariable analysis confirmed the protective role of AFDs on the incidence of AE (OR 0.089, *p* = 0.038); a pre-operative DLCO > 55% was also protective against the development of AE. Although not significant, sublobar resection and minimally invasive surgery might contribute reducing the incidence of AE; therefore, a conservative approach should be favored in IPF patients. Considering the limited cohort and few AE events, our study suggests a possible protective role for AFT (both pirfenidone and nintedanib) against post-operative AE. However, we looked at in a relatively small population of patients from three academic Italian institutions, and as such larger multicenter studies or properly designed randomized controlled trials are needed to corroborate these findings. Our study population differs from other published series with regard to ethnicity, age and us using a higher median dose of pirfenidone in our retrospective analysis.

Iwata et al. [15] demonstrated the safety and feasibility of perioperative pirfenidone therapy as well as its efficacy in preventing AE after surgery in 50 IPF Japanese patients: 90-day AE developed in 4 patients without AFT (21.1%) and in only 1 (3.2%) on-AFT patient (*p* = 0.04). This study also revealed an association between the AFT and the JACS (Japanese Association for Chest Surgeons) risk score and AE on multivariable analysis [26]. Another study on a larger cohort (*n* = 100 IPF patients) [16] showed an overall incidence of AE of 11% and 13% at 30 and 90 days; when patients were stratified by perioperative pirfenidone use, 30-day and 90-day AE developed in 3.6% and 13.9% of patients (*p* = 0.13) and 3.6% and 16.7% of patients (*p* = 0.08) in the perioperative pirfenidone group and non-pirfenidone group. A marginally significant lower incidence of AE was also reported in another retrospective study from Japan [25]. Though relatively small or retrospective, these data suggest that pirfenidone perioperative treatment could be a promising strategy in preventing AE after surgery. A prospective phase-II study [26] has confirmed the safety of pirfenidone treatment; the PIII-PEOPLE trial [27], which is ongoing, will clarify the role of perioperative pirfenidone in preventing the occurrence of AE following surgery for LC.

In both retrospective series and prospective phase II studies from Japan, the pirfenidone dose was set between 1200 mg and 1800 mg per day [15,17], whereas in our cohort, 19/24 patients (79.1%) were treated with a dose of 2403 mg per day. However, despite this higher dose, we observed no severe adverse events or delayed surgeries.

Long term outcomes following lung surgery for cancer in patients with ILD, and particularly IPF, are worse than those of patients without any ILD [9,10,11,28]. Although survival analysis was not a primary end-point of our study, we showed no differences in either OS or DFS between the AFT and non-AFT groups.

Our study has several limitations, mainly related to the rarity of the association between IPF and LC reflecting the small numbers of this study and the long study period, as well as its retrospective nature, such as the non-random allocation to the AFT group and the more recent use of prophylactic AFT. In addition, the strong association between AFT and AE resulting from the regression analysis should be considered with caution due to the few AE events. On the other hand, our study reported our real-life strategy for these frail patients, with better outcomes in those treated with AFT. We were not able to conduct a centrally controlled review of all CT scans; however, all institutions are highly experienced in ILD and we believe the possibility of misdiagnosis is low. Due to the multi-institutional nature of this study, we are unable to collect some long-term results, such as the PFT decline, the compliance and the duration of AFT and the quality of life levels. The lack of long-term evaluation of the AFT, in terms of duration, tolerability and effectiveness, could be considered another limit, but the primary aim of the study was to analyze the incidence of AE in the immediate post-operative period (90-day). Lastly, the availability of some pre-operative laboratory data was too variable between institutions, and this kind of data was excluded from the analysis.

## 5. Conclusions

In conclusion, lung cancer surgery in IPF patients is challenging and mortality is often associated with the development of AE. Our study suggests that the occurrence of these clinically relevant events may be prevented by prophylactic (or continuous) use of nintedanib or pirfenidone. Large-scale prospective studies are needed to clarify the role of perioperative AFT in reducing the risk of AE in patient with pulmonary fibrosis undergoing surgery for lung cancer.

## Figures and Tables

**Table 1:** Demographic, pre-operative, intraoperative and postoperative data and comparisons between patients without anti-fibrotic treatment and patients with anti-fibrotic treatment. (IQR: inter-quartile range; BMI: Body Mass Index; FEV1: Forced Expiratory Volume in 1 s; FVC: Forced Vital Capacity; DLCO: diffusing capacity of the lung for carbon monoxide; SpO2: Saturation of Peripheral Oxygen; ECOG PS: Eastern Cooperative Oncological Group Performance Status; CCI: Charlson Comorbidity Index; GAP: Gender-Age-Physiology; RLL: right lower lobe; LLL: left lower lobe; RM: right medium; RUL: right upper lobe; LUL: left upper lobe; ADC: adenocarcinoma; SCC: squamous cell carcinoma; SCLC: small-cell lung cancer; AE: acute exacerbation; ICU: intensive care unit; CI 95%: confidence interval 95%; DFS: disease-free survival).

Variables	No Treatment (*n* = 26)	Anti-Fibrotic Treatment (*n* = 29)	*p*-Value
Sex male, n (%)	20 (76.9)	21 (72.4)	0.76
Age in years, median (IQR)	75.5 (66–78)	72 (66–77)	0.49
BMI, median (IQR)	25 (22–28)	27 (23.21–29)	0.12
FEV1 L, median (IQR)	2.58 (2.21–2.87)	2.45 (1.94–2.91)	0.39
FEV1%, median (IQR)	96.5 (83.2–105.5)	88 (78–101)	0.21
FVC liters, median (IQR)	3.21 (2.93–3.69)	2.91 (2.3–3.69)	0.17
FVC%, median (IQR)	98 (83.2–107.2)	89 (79.5–100)	0.33
DLCO%, median (IQR)	65.5 (52.7–76)	58 (45–67)	0.063
SpO2% at rest, median (IQR)	97 (95.1–98)	95 (94–97)	0.059
ECOG PS			0.03
0	7 (26.9)	3 (10.3)	
1	16 (61.5)	13 (44.8)	
2	2 (7.7)	12 (41.4)	
3	1 (3.8)	1 (3.4)	
CCI, median (IQR)	6 (4–6.5)	5 (4–6)	0.34
GAP index, median (IQR)	3 (3–3)	3 (2–4)	0.82
Anti-fibrotic treatment, n (%)			
Chronic pirfenidone		9 (31)	
Chronic nintedanib		5 (17.3)	
Prophylactic pirfenidone		15 (51.7)	
Steroid treatment, n (%)	5 (19.2)	10 (34.5)	0.23
Concomitant emphysema, n (%)	9 (34.6)	12 (41.4)	0.78
Sublobar resection, n (%)	11 (42.3)	15 (51.7)	0.48
Minimally invasive surgery, n (%)	17 (65.4)	25 (86.2)	0.11
Location, n (%)			0.38
RLL	12 (46.2)	8 (27.6)	
LLL	5 (19.2)	7 (24.1)	
RM	3 (11.5)	3 (10.3)	
RUL	3 (11.5)	9 (31)	
LUL	3 (11.5)	2 (6.9)	
Tumor pattern CT, n (%)			1
Part-solid	5 (19.2)	5 (17.2)	
Solid	21 (80.2)	24 (82.8)	
Cinical stage, n (%)			0.76
IA	11 (42.3)	11 (37.9)	
IB	3 (11.5)	7 (24.1)	
IIA	4 (15.4)	3 (10.3)	
IIB	4 (15.4)	5 (17.2)	
IIIA	4 (15.4)	3 (10.3)	
Pathological stage, n (%)			0.77
IA	9 (34.6)	9 (31)	
IB	7 (26.9)	8 (27.6)	
IIA	1 (3.8)	0	
IIB	3 (11.5)	5 (17.2)	
IIIA	4 (19.2)	4 (13.8)	
IIIB	1 (3.8)	3 (10.3)	
Pathology, n (%)			0.64
ADC	12 (46.2)	13 (44.8)	
SCC	13 (50)	13 (44.8)	
SCLC	1 (3.8)	3 (10.3)	
Patient with at least one complication, n (%)	10 (38.5)	10 (34.5)	0.78
AE, n (%)	6 (23.1)	1 (3.4)	0.044
Clavien–Dindo classification > 3a, n (%)	8 (30.8)	7 (24.1)	0.76
Mortality, n (%)			
90 d	3 (11.5)	0	0.099
30 d	2 (7.7)	0	0.21
Operative time in minutes, median (IQR)	150 (105–200)	101 (71.5–152.5)	0.027
Estimated blood loss in mL, median (IQR)	190 (100–200)	100 (95–200)	0.1
Length of hospital stay in days, median (IQR)	7.50 (5–13)	6 (5–10)	0.17
ICU stay in days, median (IQR)	1 (0.25–1.75)	1 (0–1)	0.1
Overall survival in months (CI95%)	57 (41–72)	50 (37–63)	0.65
Disease-free survival in months (CI95%)	54 (38–60)	43 (30–57)	0.95

**Table 2:** Univariate and multivariable analysis on post-operative AE (AE: acute exacerbation; ECOG PS: Eastern Cooperative Oncological Group Performance Status; FVC: Forced Vital Capacity; DLCO: diffusing capacity of the lung for carbon monoxide; GAP: Gender-Age-Physiology; AFT: anti-fibrotic treatment).

	Univariate Analysis on AE			Multivariable Analysis on AE		
Variable	OR	CI95%	*p*	OR	CI95%	*p*
Sex male	2.22	0.24–20.3	0.47			
Age > 75 years	2.28	0.22–22.8	0.48			
FVC% > 75	0.83	0.088–7.89	0.87			
DLCO% > 55	0.23	0.046–1.21	0.085	0.19	0.032–1.14	0.07
GAP index > 4	1.52	0.25–9.02	0.64			
AFT	0.11	0.013–1.06	0.057	0.089	0.009–0.87	0.038

## Data Availability

All data are provided within the text.
